# Alterations of gut fungal microbiota in patients with rheumatoid arthritis

**DOI:** 10.7717/peerj.13037

**Published:** 2022-03-01

**Authors:** Xiaoyu Sun, Yushuang Wang, Xinke Li, Meiling Wang, Jianyi Dong, Wei Tang, Zengjie Lei, Yuling Guo, Ming Li, Yuyuan Li

**Affiliations:** 1 First Affiliated Hospital of Dalian Medical University, Dalian, Liaoning, China; 2 College of Basic Medical Science, Dalian Medical University, Dalian, Liaoning, China; 3 Dalian Municipal Central Hospital, Dalian, Liaoning, China; 4 Laboratory Animal Center, Dalian Medical University, Dalian, Liaoning, China; 5 Advanced Institute for Medical Sciences, Dalian Medical University, Dalian, Liaoning, China

**Keywords:** Rheumatoid arthritis, Dysbiosis, Gut mycobiota, Age, Gender

## Abstract

**Background:**

Rheumatoid arthritis (RA) is a systemic autoimmune disease, in addition, gut microbiota plays an important role in the etiology of RA. However, our understanding of alterations to the gut fungal microbiota in Chinese population with RA is still limited.

**Methods:**

Serum samples were obtained from 62 patients with RA, and 39 age- and gender-matched healthy controls (HCs). Fecal samples were obtained from 42 RA patients and 39 HCs. Fecal fungal microbiota targeting internal transcribed spacer region 2 (ITS2) rRNA genes was investigated using MiSeq sequencing, as well as their associations with some diagnostic biomarkers for RA.

**Results:**

Our results showed that the fungal diversity did not alter in RA patients but taxonomic composition of the fecal fungal microbiota did. The gut mycobiota of RA patients was characterized by decreased abundance of *Pholiota*, *Scedosporium*, and *Trichosporon*. The linear discriminant analysis (LDA) effect size analysis (LEfSe) analysis identified several RA-enriched fungal genera, which were positively correlated with most RA biomarkers. Furthermore, since RA is an age- and gende-related disease, we classified RA patients into subgroups with age and gender and analyzed the sequencing results. Our data demonstrated that *Wallemia* and *Irpex* were the most discriminatory against RA patients over 60 years old, while *Pseudeurotiaceae* was the most discriminatory against female RA patients.

**Conclusions:**

The case-control study presented here confirmed the alterations of gut fungal microbiota in Chinese patients with RA, and we speculated that the fungal dysbiosis may contribute to RA development.

## Introduction

Rheumatoid arthritis (RA), a persistent autoimmune disease affecting roughly 1% of the global population (Qamar, John & Bhatti, 2021), is characterized by synovial joint inflammation and chronic pain, leading to progressive disability, as well as increased morbidity and mortality (Brandl et al., 2021). The etiology of RA is multifactorial, but remains obscure. However, there is consensus that a complex interaction of genetic and environmental factors is involved in the development of RA (McInnes & Schett, 2011; Scherer, Häupl & Burmester, 2020). As an indispensable environmental factor, gut microbiota has been more appreciated in recent studies on the progression of RA (Belkaid & Hand, 2014; Horta-Baas et al., 2017; Sun et al., 2019; Wells et al., 2020). Significant alterations in the composition of gut microbiota, namely dysbiosis, have been associated with RA. It is thought that the overgrowth of Gram-negative bacteria in the gut can cause an increase in toxic metabolites, which enter the bloodstream and eventually lead to arthritis. A Chinese study has shown that early RA patients had significantly more *Lactobacillus* communities than healthy individuals, suggesting an alteration of *Lactobacillus* communities in the early state of RA (Liu et al., 2013). Scher et al. (2013) found that new-onset untreated RA (NORA) patients in American population had an increased abundance of *Prevotella copri* (*P. copri*) and a reduced abundance of *Bacteroides* species in the intestine. In contrast, *Faecalibacterium*, which is generally recognized as a beneficial microbe, decreased in RA patients. In a separate study, *Haemophilus* species were also found to be depleted in RA patients at all three sites, that is, saliva, dental plaque, and subgingival plaque (Chu et al., 2021). These findings indicate that RA is associated with dysbiosis of the gastrointestinal tract.

In addition, other types of microorganisms, such as fungi, viruses and archaea, also colonize the gastrointestinal tract and play pivotal roles in various activities such as immune response, mucosal barrier and energy metabolism (Li et al., 2018). In particular, fungi have drawn recent attention as important microorganisms that maintain gut homeostasis in humans and animals, representing 0.1–1.0% of the intestinal microbiota (commonly referred to as mycobiota) (Auchtung et al., 2018). Actually, mycobiota has long been strongly linked to autoimmune diseases in people with certain genetic backgrounds and environmental factors, although their compositions have not been thoroughly examined. As an illustration, *Candida* (mainly *C. albicans*) is the predominant genus reproducibly identified in fecal samples from IBD cohorts (Sokol et al., 2017; Iliev & Cadwell, 2021). Our previous study discovered a mycobiota dysbiosis in ankylosing spondylitis (AS) patients, with higher levels of *Ascomycota* and lower levels of *Basidiomycota* (Li, Leonardi & Iliev, 2019). Hammad, Liyanapathirana & Tonge, 2019 reported that fungal DNA in the synovial fluid of RA patients was characterized by increased abundance of *Ascomycota* and decreased abundance of *Basidiomycota*. Well-designed studies are required, as well as an in-depth characterization of the fecal fungi of RA patients.

In this study, we characterized the fungal microbiota in stool samples from Chinese patients with RA using internal transcribed spacer 2 (ITS2) region sequencing. We also compared the gut mycobiota of RA patients with different disease activity parameters, including inflammatory markers (C-reactive protein (CRP) and erythrocyte sedimentation rate (ESR)), rheumatoid factor (RF), immunoglobulin G (IgG), immunoglobulin A (IgA), immunoglobulin M (IgM), immunoglobulin E (IgE), and also tumor markers (carcinoembryonic antigen (CEA), carbohydrate antigen (CA)19-9 (CA19-9), CA125). Our study represented the first analysis of fecal fungal dysbiosis in Chinese RA patients, and the data provide a basis for possible specific therapeutic interventions for RA.

## Materials and Methods

### Ethics statement

This study protocol was approved by the Ethics Committees of Dalian Municipal Central Hospital, Dalian, China (YN2021-003-01). All the procedures were performed in accordance with the guidelines approved by the Ethics Committee of Dalian Medical University, China. After receiving a written description of the aim of this study, all subjects signed written informed consent prior to study enrollment.

### Subject recruitment and sample collection

From January 2020 to February 2021, a total of 62 patients with RA were recruited from Dalian Municipal Central Hospital in Dalian, China, and 29 healthy individuals also agreed to participate. RA patients fulfilled the RA criteria of the American College of Rheumatology (ACR)/European League Against Rheumatism (EULAR) 2010. Participants were excluded if they had taken antibiotics, probiotics or prebiotics within the previous 3 months, or had a history of inflammatory bowel disease or other autoimmune diseases, or gastrointestinal tract disorders. Fecal samples obtained from 42 RA patients and 39 healthy controls (HCs) were transported in liquid nitrogen and stored at −80 °C until fungal DNA was extracted. All serum samples were stored at −80 °C prior to use. Serum CRP, ESR, RF, IgG, IgA, IgM, IgE, CEA, CA19-9, and CA125 were measured on the day of stool sample collection in the laboratory at Dalian Municipal Central Hospital. An immunoturbidimetric assay was used to determine the values of serum CRP, RF, IgG, IgA, IgM, and IgE (Beckman Coulter, Brea, CA, USA). Serum CEA, CA19-9, and CA125 concentrations were determined by electrochemiluminescence (ECL) (Roche; Elecsys, Lenexa, KS, USA), and ESR was determined using an automatic erythrocyte sedimentation analyzer.

### Genomic DNA extraction, PCR amplification and ITS2 gene sequencing

In accordance with the manufacturer protocol previously described, total metagenomic DNA in 200 mg (fresh weight) of each fecal sample was extracted using the QIAamp DNA stool minikit (Qiagen, Hilden, Germany) (Li, Leonardi & Iliev, 2019). The concentration and quality of DNA were measured by Nanodrop2000 (Thermo Fisher Scientific, Wilmington, CA, USA) and Agar Gel electrophoresis, respectively. The fungal microbiome was analyzed through the sequencing of internal transcribed spacer region 2 (ITS2). An ITS2 fragment (approximately 320 bases) was amplified with primers ITS3F (5′-GCATCGATGAAGAACGCAGC-3′) and ITS4R (5′-TCCTCCGCTTATTGATATGC-3′). The thermal cycle conditions were: initial denaturation at 98 °C for 2 min, followed by 25 cycles, denaturation at 98 °C for 15 s, annealing at 55 °C for 30 s, extension at 72 °C for 30 s, and extension at 72 °C for 5 min. After the individual quantification step, amplicons were pooled in equal amounts, and pair-end 2 × 300 bp sequencing was performed using the Illlumina MiSeq platform with MiSeq Reagent Kit v3 at Shanghai Personal Biotechnology Co. Ltd. (Shanghai, China).

### Sequence analysis

The Quantitative Insights into Microbial Ecology (QIIME, v1.8.0) pipeline was employed to process the sequencing data, as previously described (Caporaso et al., 2010). Raw sequencing reads with exact matches to the barcodes were assigned to respective samples and identified as valid sequences. The low-quality sequences were screened using the following criteria (Gill et al., 2006; Chen & Jiang, 2014): sequences with a length less than 150 bp, sequences with mean Phred score less than 20, sequences with fuzzy bases, and single nucleotide repeats with a length greater than 8 bp. After chimera detection, the remaining high-quality sequences were clustered into operational taxonomic units (OTUs) with 97% sequence homology by UCLUST (Edgar, 2010). A representative sequence was selected from each OTU using default parameters. OTU taxonomic classification and OTU table generation was conducted as previously described (Zheng et al., 2020).

### Bioinformatic analysis

The sequence data analysis was mainly performed using QIIME and R packages (v3.2.0). The community richness and diversity (alpha diversity) analysis (Chao1 and Shannon) was performed using QIIME 2 with the same sequence depth. The beta diversity analysis, principal coordinate analysis (PCoA), weighted or unweighted UniFrac analysis by R software were used to investigate the structural variation of microbial communities across samples. The linear discriminant analysis (LDA) effect size analysis (LEfSe) was performed to detect differentially abundant taxa across groups using the default parameters. The correlations between laboratory markers (including RF, CRP, IgG, IgA, IgM, IgE, CEA, CA19-9, CA125 and ESR) and fungal genera in fecal samples from RA patients were determined using Spearman’s method. A partial least squares discriminant analysis (PLS-DA) with a variable importance in projection (VIP) plot (Yuan et al., 2018) was performed to determine possible differences in OTUs between groups, which would help predict the functional contents of the metagenome. Key genera of VIP > 1.8 were considered important contributors to the model.

### Statistical analysis

All data were presented as arithmetic mean ± standard error of mean (SEM). With the assistance of GraphPad Prism 7 (Graph Pad Software, La Jolla, CA, USA), the comparison between the two groups was statistically analyzed using the nonparametric t test, and multiple comparison adjustments were made with the FDR algorithm (Benjamini et al., 2001). Spearman’s rank correlation test was used to perform a correlation analysis. The statistical analysis was performed using SPSS version 19.0 (SPSS Inc., Chicago, IL, USA). Results were considered to be statistically significant with *P* < 0.05.

### Accession number

The sequence data from this study were deposited in the NCBI Sequence Read Archive with the accession number RPJNA735021.

## Results

### Investigation of clinical characteristics of RA patients and healthy controls

A total of 101 individuals were included in the current analysis. The clinical characteristics of all subjects are described in Table 1. The 62 (15 male, 47 female) patients with RA ranged in age from 33 to 80 years, with a mean of 60.8 years. The 39 (7 male, 32 female) healthy controls ranged in age from 32 to 80 years, with a mean of 60.4 year. Given that RA is three times more common in women than men (Favalli et al., 2019), there were more women than men in this study, which was consistent with the previous study (Sun et al., 2019). RA patients had higher CRP, ESR, RF, IgA, IgE, and CA125 levels than healthy controls, but there were no significant differences in other general biological indexes, such as IgG, IgM, CEA, and CA19-9 levels between the two groups (Table 1). RA patients were classified into subgroups by age and gender (Table 2), and the results showed that the levels of CRP and CEA in patients over 60 years old appeared increased compared with those under 60 years of age, while the level of IgM was decreased significantly in the older population (Table 2). However, these indexes showed no significant difference between the healthy controls when stratified by age (Table 3). The levels of IgA and IgE in male patients were significantly higher than those of female patients (Table 2), while IgE levels in the male control group showed a downward trend, but the difference was not statistically significant (Table 3).

**Table 1:** Clinical characteristics of subjects.

	RA (*n* = 62)	HC (*n* = 39)	*P* value
Male/Female (N)	15/47	7/32	
Age (Mean ± SEM)	60.80 ± 1.33	60.44 ± 1.47	0.87
CRP (mg/L)	12.54 ± 1.73	6.29 ± 2.42	0.039*
ESR (mm/h)	39.10 ± 2.84	14.95 ± 2.28	<0.0001****
RF (IU/mL)	362.93 ± 57.09	20.60 ± 0.36	<0.0001****
IgG (IU/mL)	1,426.38 ± 46.5	1,394.51 ± 83.38	0.738
IgA (IU/mL)	314.34 ± 16.75	235.62 ± 17.15	0.003**
IgM (IU/mL)	113.12 ± 6.46	147.51 ± 24.51	0.12
IgE (IU/mL)	94.63 ± 14.88	49.13 ± 7.24	0.03*
CEA (ng/mL)	2.18 ± 0.15	2.39 ± 0.27	0.48
CA19-9 (IU/mL)	13.42 ± 1.70	14.90 ± 1.45	0.561
CA125 (IU/mL)	17.98 ± 1.96	10.37 ± 0.67	0.015*

**Notes:**

* *P* < 0.05.

** *P* < 0.01.

**** *P* < 0.0001.

**Table 2:** Clinical characteristics of the RA group.

	Under 60 (*n* = 24)	Over 60 (*n* = 38)	*P* value
Male/Female (N)	6/18	9/29	
Age (Mean ± SEM)	50.50 ± 1.51	67.26 ± 0.97	0.013*
CRP (mg/L)	7.81 ± 1.63	15.74 ± 2.54	0.03*
ESR (mm/h)	36.30 ± 4.52	40.89 ± 3.67	0.447
RF (IU/ml)	348.82 ± 78.11	379.32 ± 81.79	0.781
IgG (IU/ml)	1,491.00 ± 52.4	1,392.37 ± 66.74	0.33
IgA (IU/ml)	338.25 ± 27.57	301.06 ± 21.11	0.31
IgM (IU/ml)	133.80 ± 12.03	102.24 ± 6.97	0.02*
IgE (IU/ml)	96.31 ± 24.79	93.61 ± 18.84	0.93
CEA (ng/ml)	1.69 ± 0.14	2.46 ± 0.21	0.016*
CA19-9 (IU/ml)	9.97 ± 1.89	15.43 ± 2.45	0.14
CA125 (IU/ml)	13.51 ± 0.91	20.37 ± 3.01	0.169
	**Male (*n* = 15)**	**Female (*n* = 47)**	***P* value**
Age (Mean ± SEM)	60.00 ± 2.69	61.02 ± 1.55	0.746
CRP (mg/L)	13.77 ± 3.07	12.18 ± 2.07	0.715
ESR (mm/h)	40.29 ± 4.14	38.73 ± 3.52	0.823
RF (IU/ml)	544.68 ± 179.55	303.99 ± 47.74	0.11
IgG (IU/ml)	1,498.00 ± 86.92	1,401.40 ± 54.91	0.384
IgA (IU/ml)	381.00 ± 33.99	292.12 ± 18.35	0.028*
IgM (IU/ml)	112.00 ± 13.82	113.51 ± 7.37	0.922
IgE (IU/ml)	170.78 ± 43.89	72.63 ± 12.79	0.006**
CEA (ng/ml)	2.05 ± 0.15	2.22 ± 0.19	0.637
CA19-9 (IU/ml)	8.76 ± 1.15	14.94 ± 2.18	0.136

**Notes:**

* *P* < 0.05.

** *P* < 0.01.

**Table 3:** Clinical characteristics of the HC group.

	Under 60 (*n* = 14)	Over 60 (*n* = 25)	*P* value
Male/Female (N)	3/11	6/19	
Age (Mean ± SEM)	50.79 ± 1.73	65.84 ± 1.01	<0.0001****
CRP (mg/L)	2.62 ± 0.96	8.43 ± 3.73	0.259
ESR (mm/h)	9.92 ± 1.81	17.56 ± 3.28	0.447
RF (IU/ml)	20.00 ± 0	20.98 ± 0.57	0.21
IgG (IU/ml)	1,418.77 ± 79.49	1,381.38 ± 122.92	0.84
IgA (IU/ml)	254.69 ± 35.30	225.29 ± 18.48	0.43
IgM (IU/ml)	132.61 ± 21.75	155.58 ± 36.34	0.67
IgE (IU/ml)	60.61 ± 25.78	94.53 ± 38.68	0.56
CEA (ng/ml)	2.67 ± 0.69	2.25 ± 0.22	0.49
CA19-9 (IU/ml)	13.13 ± 2.40	15.79 ± 1.83	0.41
CA125 (IU/ml)	11.90 ± 0.89	9.61 ± 0.88	0.18
	**Male (*n* = 7)**	**Female (*n* = 32)**	***P* value**
Age (Mean ± SEM)	64.75 ± 2.94	59.40 ± 1.70	0.15
CRP (mg/L)	8.75 ± 7.28	5.79 ± 2.53	0.63
ESR (mm/h)	9.12 ± 3.63	17.00 ± 2.72	0.17
RF (IU/ml)	20.51 ± 0.51	20.64 ± 0.46	0.889
IgG (IU/ml)	1,036.71 ± 73.35	1,476.90 ± 99.90	0.045*
IgA (IU/ml)	162.00 ± 16.00	253.13 ± 20.50	0.044*
IgM (IU/ml)	86.57 ± 13.94	163.79 ± 30.80	0.24
IgE (IU/ml)	29.77 ± 9.69	98.04 ± 33.52	0.33
CEA (ng/ml)	3.30 ± 0.74	2.10 ± 0.28	0.08
CA19-9 (IU/ml)	16.54 ± 3.62	14.37 ± 1.64	0.566

**Notes:**

* *P* < 0.05.

**** *P* < 0.0001.

### Comparison of mycobiota between RA patients and healthy controls

**Figure 1:**
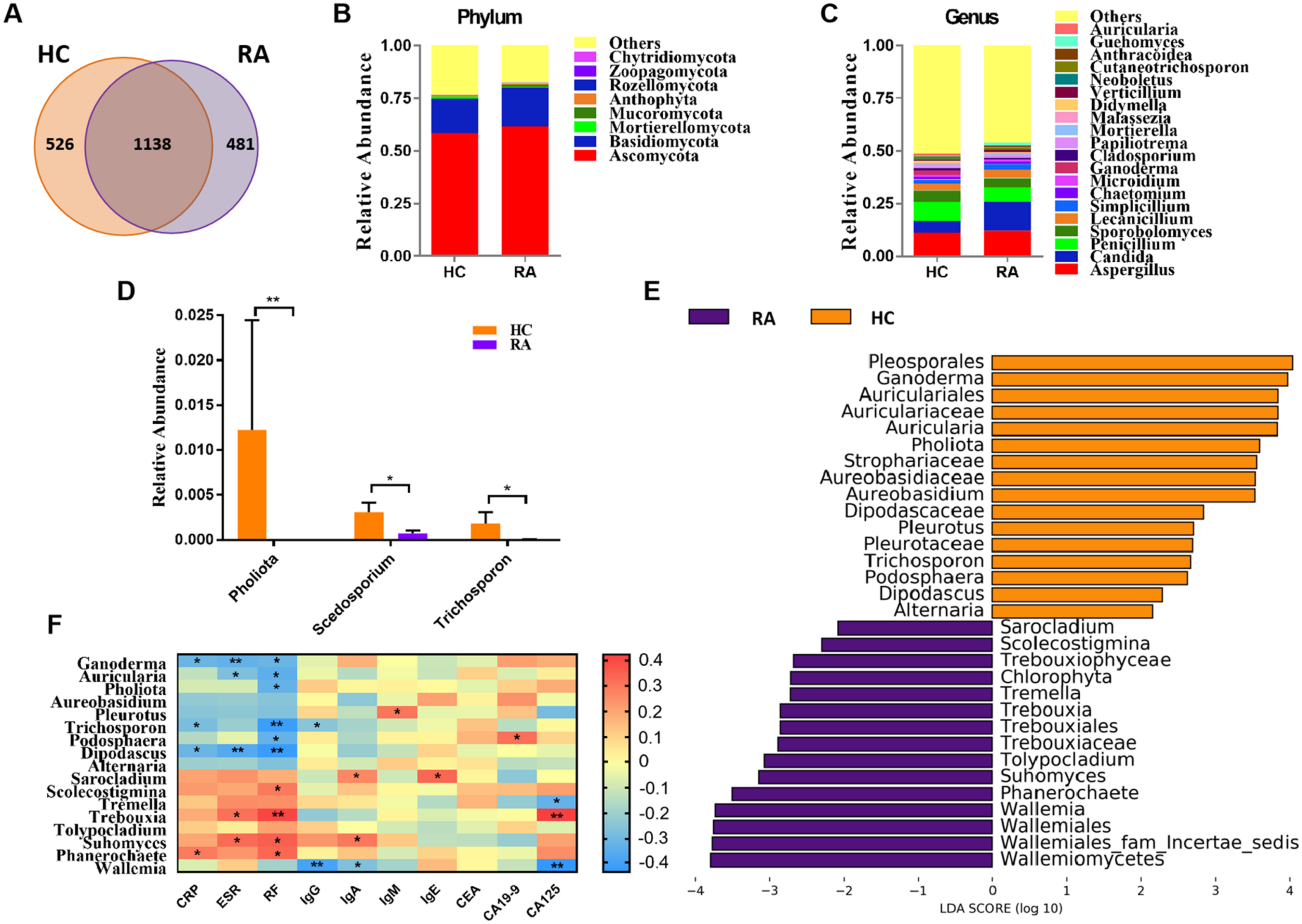
Comparison of mycobiota between RA patients and healthy controls. (A) Venn diagram of shared and independent fungal OTUs between the two groups; Fungal community bar plot at the phylum (B) and genus (C) levels; (D) the analysis of species difference at genus level with the values evaluated as mean ± SEM; (E) LEfSe analysis of mycobiota between the RA patients and healthy controls; (F) Heatmap of Spearman correlations between fungal taxa and serological features of RA. The color red represents the highest score value (the highest correlation), while blue marks the lowest score value (the lowest correlation). *P < 0.05, **P < 0.01.

In the present study, we obtained a total of 4,457,120 high-quality filtered reads from the fecal mycobiota of 39 HCs (48,412 ± 14,456) and 42 RA patients (61,167 ± 19,134) for subsequent analysis, with an average of 54,789 ± 18,112 reads per sample. The results showed that there were 526 OTUs unique to the HC group and 481 OTUs unique to the RA group, with a total of 1,138 OTUs in the two groups (Fig. 1A). Compared with HCs, the community richness (assessed using Chao1) and alpha diversity (assessed using the Shannon index) of intestinal fungi in RA patients was relatively decreased, but there were no statistical differences between both indexes (Figs. S1A and S1B, all *P* > 0.05).

Based on the Bray-Curtis, Jaccard, and Unweighted UniFrac algorithms, we then evaluated and compared the beta diversity of fungal flora in RA patients and healthy controls. The RA and healthy control groups could not be divided into different clusters (Adonis test, *P* > 0.05; Fig. S1C). Taken together, the alpha- and beta-diversity analyses demonstrated no significant changes in fungal community structure in RA patients compared with healthy controls. The analysis of the relative levles of fungi (Fig. 1B) showed that the *Ascomycota* and *Basidiomycota* phyla were the dominant taxa in both the HC and RA groups. However, there was no obvious change in the proportions of these two phyla in RA patients. At the genus level, classified genera including *Aspergillus*, *Candida*, and *Penicillium* were abundant in the fungal microbiota of both RA patients and healthy controls. Among the most dominant genera, *Candida* was increased in RA patients, but this increase did not reach statistical significance (*P* = 0.07, Fig. 1C). We found that the levels of *Pholiota* (*P* = 0.006), *Scedosporium* (*P* = 0.042), and *Trichosporon* (*P* = 0.037) in the RA group were lower than the HC group at the genus level (Fig. 1D).

LEfSe analysis was further performed to identify fungal groups with significant differences in levels between the RA and HC groups (LDA score > 2, *P* < 0.05; Fig. 1E). The comparison of the RA and HC groups revealed that most of the differential fungi could be classified at the genus level. *Ganoderma*, *Auricularia*, *Pholiota*, *Aureobasidium*, *Pleurotus*, *Trichosporon*, *Podosphaera*, *Dipodascus*, and *Alternaria* were enriched in the healthy controls, whereas *Wallemia*, *Phanerochaete*, *Suhomyces*, *Tolypocladium*, *Trebouxia*, *Tremella*, *Scolecostigmina*, and *Sarocladium* were prevalent in RA patients. Although most of these different genera were not abundant, our results suggested that fungal dysbiosis occurred in RA patients.

The Spearman correlation between mycobiota and physiological measures of RA was analyzed as well (Fig. 1F). The results indicated that the abundance of the vast majority of mycobiota prevalent in the healthy controls was negatively correlated with some serological features of RA; in contrast, the enrichment in RA patients was positively correlated with some serological characteristics of RA, except *Wallemia*, which was negatively related to the serum levels of IgG, IgA and CA125. As shown in Fig. S1D, the PLS-DA score plots showed model discrimination between RA patients and healthy controls. A total of three genera were recorded with VIP > 1.8 (Fig. S1E).

### Comparison of mycobiota between RA patients aged less than 60 and over 60 years old

**Figure 2:**
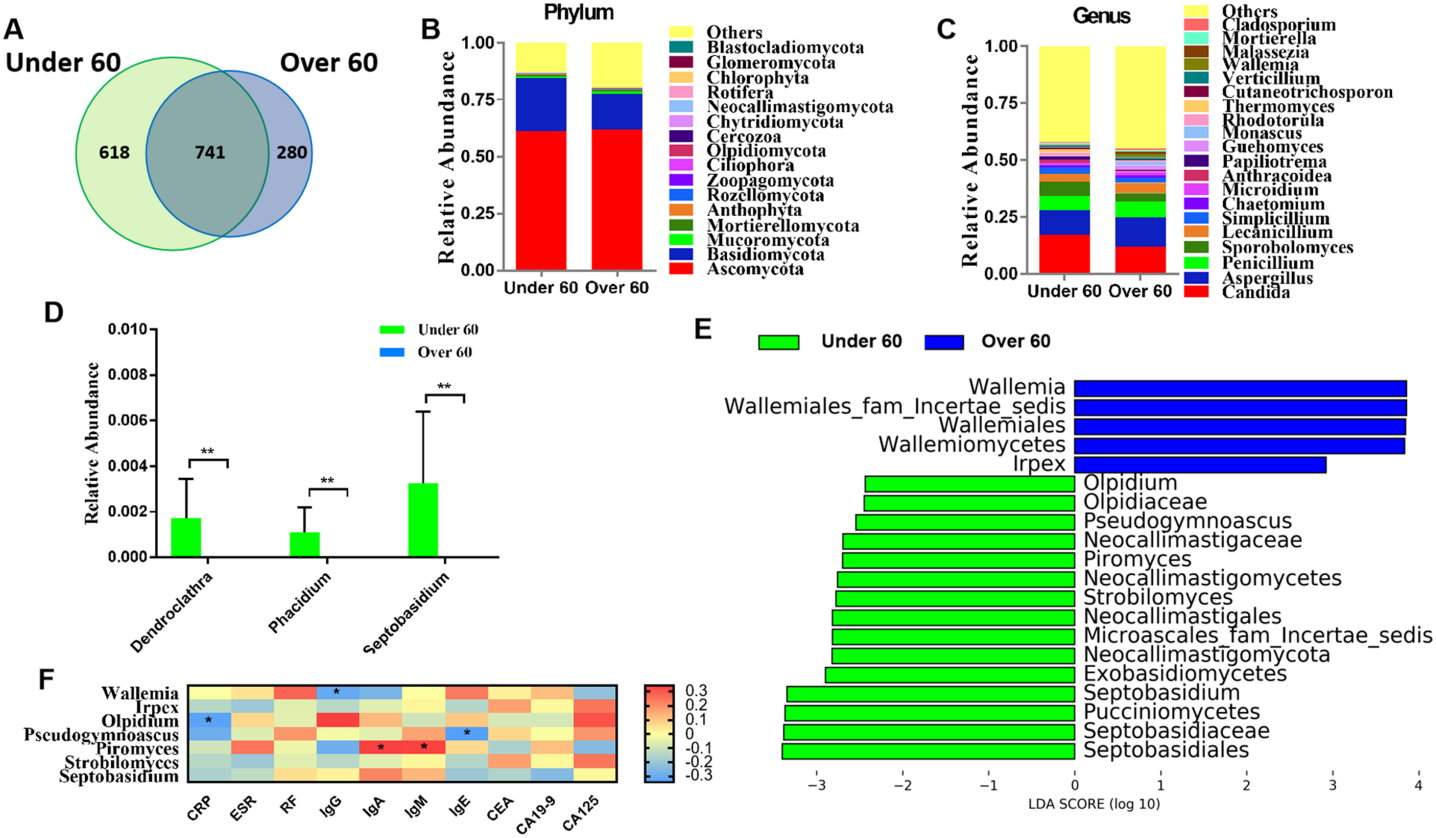
Comparison of mycobiota between RA patients aged less than 60 and those over 60 years of age. (A) Venn diagram of shared and independent fungal OTUs between the two groups; fungal community bar plot at the phylum (B) and genus (C) levels; (D) the analysis of species difference at the genus level with the values evaluated as mean ± SEM; (E) LEfSe analysis of mycobiota between the RA patients aged under 60 and over 60 years; (F) Heatmap of Spearman correlations between fungal taxa and serological features of RA. The color red represents the highest score value (the highest correlation), while blue marks the lowest score value (the lowest correlation). *P < 0.05, **P < 0.01.

RA is an age-related disease, and RA patients were classified into two subgroups: those over 60 years old and those aged under 60. Sequencing results showed that there were 618 OTUs unique to the under 60 group and 280 OTUs unique to the over 60 group, with a total of 741 OTUs in the two groups (Fig. 2A). The calculated fungal richness and alpha-diversity indices showed no significant changes between the two groups (Figs. S2A and S2B, all *P* > 0.05). An analysis of beta diversity did not show a distinct clustering of samples in the under 60 and over 60 groups (Adonis test, *P* > 0.05; Fig. S2C). These data suggested no difference in the fungal composition of RA patients of different ages. The compositions of the fungal microbiota in RA patients under 60 years old and over 60 years old were assessed at the phylum and genus levels (Figs. 2B and 2C). We found that, at the genus level, the abundances of *Dendroclathra* (*P* = 0.009), *Phacidium* (*P* = 0.009), and *Septobasidium* (*P* = 0.009) in the Over 60 group were lower than those in the under 60 group (Fig. 2D). LEfSe analysis revealed *Wallemia* and *Irpex* as the most able to identify RA patients over 60 years old. The fungal microbiota that was able to distinguish RA patients under 60 years old were *Olpidium*, *Pseudogymnoascus*, *Piromyces*, *Strobilomyces*, and *Septobasidium* (Fig. 2E). Spearman correlation analysis demonstrated that *Wallemia* was negatively related to the serum level of IgG, *Olpidium* was negatively related to the serum level of CRP, *Pseudogymnoascus* was negatively related to the serum level of IgE, while *Piromyces* was positively related to the serum levels of IgA and IgM (Fig. 2F). PLS-DA score plot exhibited model discrimination between RA patients aged under 60 and over 60 years (Fig. S2D). A total of five genera were recorded with VIP > 1.8 (Fig. S2E).

### Comparison of mycobiota between male and female RA patients

**Figure 3:**
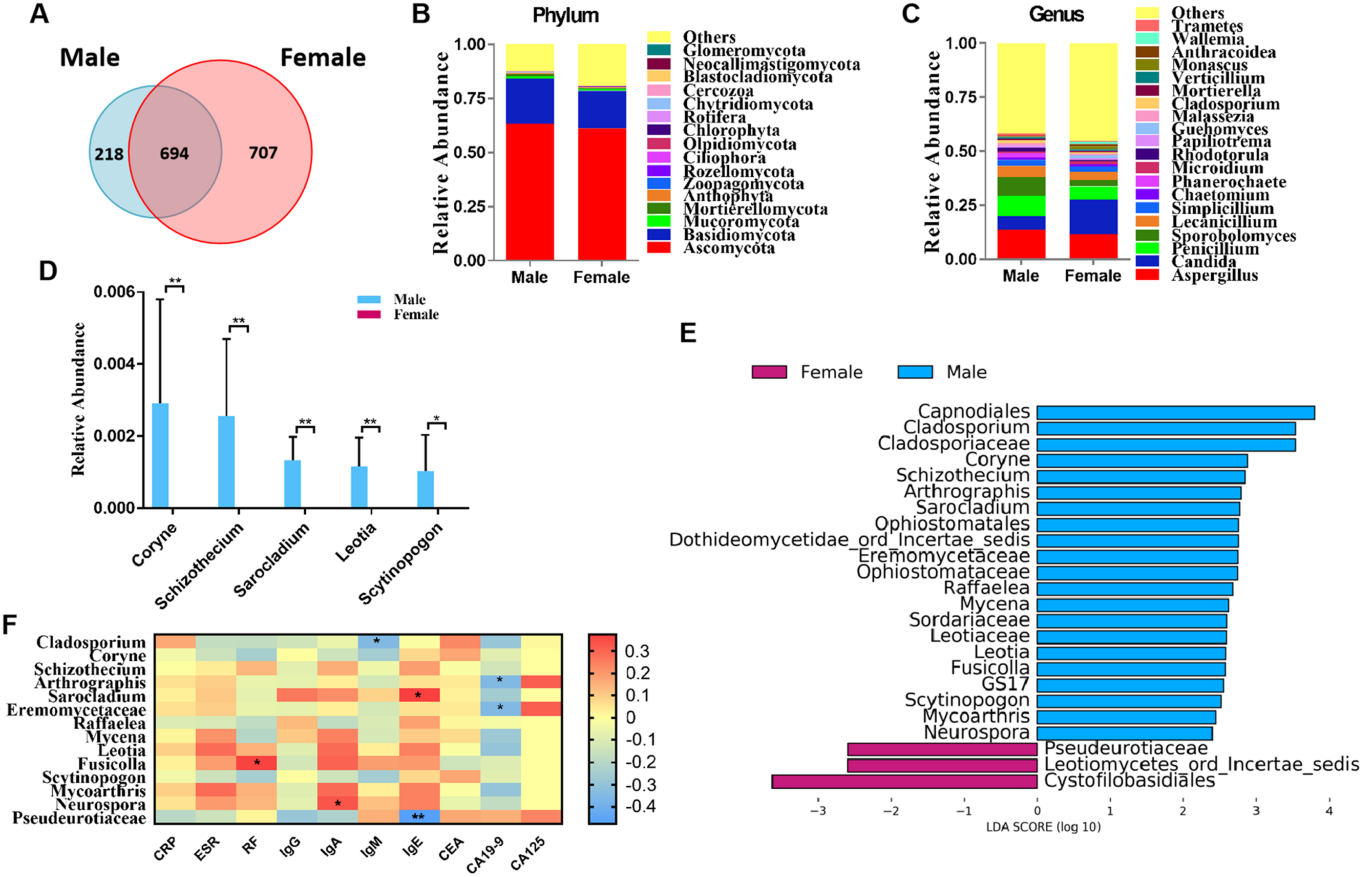
Comparison of mycobiota between male and female RA patients. (A) Venn diagram of shared and independent fungal OTUs between the two groups; fungal community bar plot at the phylum (B) and genus (C) levels; (D) the analysis of species difference at the genus level with the values evaluated as mean ± SEM; (E) LEfSe analysis of mycobiota between the male and female RA patients; (F) Heatmap of Spearman correlations between fungal taxa and serological features of RA. The color red represents the highest score value (the highest correlation), while blue marks the lowest score value (the lowest correlation). *P < 0.05, **P < 0.01.

We classified RA patients into subgroups by gender and analyzed the sequencing results. In total, 694 OTUs overlapped between the male and female RA patients, in addition, 218 and 707 OTUs were uniquely present in the male and female RA patients, respectively (Fig. 3A). Analysis of alpha and beta diversities showed no significant changes between the two groups, suggesting no significant difference in fungal composition between female patients and male patients (Figs. S3A–S3C). The compositions of the fungal microbiota in male and female RA patients were analyzed at the phylum and genus levels (Figs. 3B and 3C). We found that the levels of *Coryne* (*P* = 0.006), *Schizothecium* (*P* = 0.006), *Sarocladium* (*P* = 0.006), *Leotia* (*P* = 0.006) and *Scytinopogon* (*P* = 0.044) were lower in female patients than in male patients at the genus level (Fig. 3D). LEfSe analysis revealed *Pseudeurotiaceae* was the most identifiable among female RA patients; while the fungal microbiota most able to identify male patients with RA were: *Cladosporium*, *Coryne*, *Schizothecium*, *Arthrographis*, *Sarocladium*, *Eremomycetaceae*, *Raffaelea*, *Mycena*, *Leotia*, *Fusicolla*, *Scytinopogon*, *Mycoarthris*, and *Neurospora* (Fig. 3E). Spearman correlation analysis demonstrated that *Pseudeurotiaceae* was negatively related to the serum level of IgE, *Cladosporium* was negatively related to the serum level of IgM, *Arthrographis* and *Eremomycetaceae* were negatively related to the serum level of CA19-9, while *Fusicolla* was positively related to the serum levels of RF, *Sarocladium* was positively related to the serum levels of IgE, and *Neurospora* was positively related to serum levels of IgA (Fig. 3F). A PLS-DA score plot revealed a clear discrimination between the microbial profile of female RA patients compared with male RA patients (Fig. S3D). Associated VIP scores (>1.8) allowed ranking of key microbial genera based on their importance in discriminating between female and male RA patients (Fig. S3E).

## Discussion

The pathogenesis of RA remains unknown; however, it is now generally accepted that microbial factors are involved. A slew of different gut bacteria have been extensively investigated as potential culprits (Brandl et al., 2021; Wu et al., 2016). Unlike the bacterial community, the composition and diversity of fungal microbiota remain largely rudimentary (Scanlan & Marchesi, 2008). Mycobiota continues to be a key component of the microbiota in the human gastrointestinal tract and has a profound influence on modulating local as well as peripheral immune responses (Iliev & Leonardi, 2017; Li, Leonardi & Iliev, 2019). Nevertheless, in Chinese RA patients, the details of the mycobiota remain unclear, therefore, in the present study, we recruited 101 individuals to study the characteristics of their gut mycobiota composition. Through high-throughput sequencing analysis of the ITS2 marker gene, we found that alterations to several key differential fungi were associated with RA and had clear correlations with RA seropositivity, suggesting a potential role of fungi in RA pathogenesis.

Fungal DNA was identified in 67% (42/62) of the RA samples and 100% (39/39) of the healthy controls. At the phylum level, the intestinal fungi was predominated by two key phyla, namely, *Ascomycota* and *Basidiomycota*, further supporting the notion of core intestinal fungi (Fig. 1B) (Nash et al., 2017; Hallen-Adams & Suhr, 2017). Among the most dominant genera, *Candida* exhibited an increasing trend in RA patients accounting for 5.5% and 13.8% of HC and RA donor fungal sequences, respectively (Fig. 1C). *Candida* is the most implicated fungal pathogen in various autoimmune diseases, such as psoriasis, Crohn’s disease, rheumatoid arthritis, psoriatic arthritis, ankylosing spondylitis, non-infectious uveitis, and multiple sclerosis (Patel & Kuchroo, 2015). Following a statistical analysis, we found the levels of *Pholiota*, *Scedosporium*, and *Trichosporon* were significantly different in their abundance between the two groups (Fig. 1D). The genus *Pholiota* (*Strophariaceae*, *Basidiomycota*) is made up of wood-rotting saprotrophic mushrooms (Lee et al., 2020), and *Pholiota* species exhibit prominent anti-tumor, antioxidant, anti-inflammatory and antinociceptive effects (Zou et al., 2019; Chou et al., 2019; Abreu et al., 2019). The genara *Scedosporium* and *Trichosporon* have been increasingly recognized as important agents of opportunistic systemic infections, mainly in immunocompromised patients (Mouhajir et al., 2020; Cordeiro et al., 2019). Immunoglobulin content was reduced in RA patients compared with HC, which may be related to the disease status of RA patients who did not receive drug therapy. Furthermore, LEfSe analysis identified that both *Pholiota* and *Trichosporon* were prevalent in the healthy controls, which was in line with the statistical analysis (Fig. 1E).

The diagnosis of RA relies on both physical examination and laboratory blood testing. The CRP and ESR are important disease activity biomarkers of RA (George et al., 2017). RF, a class of immunoglobulins (Igs), is found in 50% of early RA patients rising to 90% at the advanced stages of the disease (Ingegnoli, Castelli & Gualtierotti, 2013; Arntz et al., 2018). Due to the increased risk of certain cancers (De Cock & Hyrich, 2018; Simon et al., 2019), tumor markers (CEA, CA19-9, and CA125) are diagnostic biomarkers for RA (Zheng et al., 2021). The results of our study showed that CRP, ESR, RF, IgA, IgE, and CA125 levels in RA patients were significantly higher than those in healthy controls (Table 1). Interestingly, the levels of the vast majority of mycobiota prevalent in RA patients were found positively correlated with these serological characteristics of RA. By contrast, the enrichment in the healthy controls was inversely associated with these serological features of RA, indicating that specific fungi may contribute to RA symptoms. It is worth noting that *Wallemia*, abundant in RA patients, was negatively related to the serum levels of IgG, IgA and CA125. *Wallemia* spp. are commonly found as a minor component of commensal gastrointestinal mycobiota in both humans and mice, though its metabolic implications in the mammalian intestine are still unknown. Some studies have reported an association between *Wallemia* exposure and asthma, suggesting that *Wallemia* may alter the pulmonary immune responses (Wheeler et al., 2016; Skalski et al., 2018). However, Imai et al. (2019) reported that the frequency of the genus *Wallemia* in patients with Crohn’s disease was significantly lower than in healthy controls. Although Crohn’s disease and RA are both autoimmune diseases, the changes seen in the frequency of *Wallemia* are different, possibly indicating a complex role of the genus *Wallemia* that requires further study.

RA occurs most frequently in middle-aged and elderly persons (Lin et al., 2015). In the present study, the number of patients aged over 60 years old was 1.5 times that of patients under 60 years old, and CRP and CEA levels in patients over 60 years old were significantly higher than those under 60 years old. Using high-throughput sequencing techniques, we found that at the genus level, the frequencies of the genera *Dendroclathra*, *Phacidium*, and *Septobasidium* were lower in the over 60 group than in the under 60 group (Fig. 2D), though the importance of this difference still remains unclear. *Wallemia* is prevalent in RA patients, especially among those over 60 years old (Fig. 2E). This reminds us that the overgrowth of *Wallemia* is one of the concerns for the development of RA. Although the importance of the other fungal genera remains unclear, they may represent some aspects of fungal dysbiosis in RA patients aged over 60 years old.

Gender is the main risk factor for RA (Alamanos & Drosos, 2005), moreover, the morbidity of RA is higher in women than in men (van Vollenhoven, 2009). In the present study, the number of female patients was three times that of male patients, and the IgA and IgE levels of female patients were significantly lower than those of male patients (Table 2). However, there was no significant difference in IgA and IgE levels in female patients compared with healthy female controls, while IgA and IgE levels in male patients were significantly higher than healthy male controls. Heavy alcohol consumption has been reported to be associated with higher IgA and IgE concentrations (Gonzalez-Quintela et al., 2008). It is widely believed that people with chronic arthritis take alcohol because of its analgesic effects (Bradlow & Mowat, 1985). Therefore, elevated IgA and IgE levels in men with RA may be associated with alcohol consumption. Previous studies demonstrated that sex hormones can induce autoimmune diseases by activating the development and activity of the immune system (Ngo, Steyn & McCombe, 2014; Cutolo & Straub, 2020). Multiple microbiota and their metabolites have the ability to affect the endocrine system and steroid hormones (Rizzetto et al., 2018), which may play a role in RA. We identified that the specific fungal genus *Pseudeurotiaceae* was the most abundant fecal fungal microbiota in female RA patients compared with male RA patients. Although the importance of this fungal genus remains unclear, it may represent some aspects of fungal dysbiosis in female patients with RA.

In conclusion, the data reported here extended our understanding of the mycobiota composition of Chinese RA patients. Although there was no significant change in fecal fungal diversity between RA patients and healthy controls, the composition of fungal microbiota changed significantly. The more precise evaluation of the role of intestinal fungi in the pathogenesis of RA, as well as its potential clinical utility as a therapeutic target, need to be further studied.

## Supplemental Information

10.7717/peerj.13037/supp-1 Supplemental Information 1 Raw data for Tables.

10.7717/peerj.13037/supp-2 Supplemental Information 2 Comparison of mycobiota between RA patients and health controls. Comparison of the Chao1 (A), and Shannon (B) index of two groups; Principal coordinate analysis (PCoA) plots of individual fungal microbiota based on unweighted (C) Unifrac distances in the RA patients and the healthy controls; (D) The PLS-DA score plots showing model discrimination between RA and HC groups; (E) The VIP plot indicating the most discriminating fungal taxa in the descending order of importance. The colored boxes on the right indicate the relative amount of the corresponding taxa in each group.

10.7717/peerj.13037/supp-3 Supplemental Information 3 Comparison of mycobiota between RA patients aged less than 60 and those over 60 years of age. Comparison of the Chao1 (A), and Shannon (B) index of two groups; Principal coordinate analysis (PCoA) plots of individual fungal microbiota based on unweighted (C) Unifrac distances in the RA patients aged less than 60 and over 60 years; (D) The PLS-DA score plots showing model discrimination between RA patients aged less than 60 and over 60 years; (E) The VIP plot indicating the most discriminating fungal taxa in the descending order of importance. The colored boxes on the right indicate the relative amount of the corresponding taxa in each group.

10.7717/peerj.13037/supp-4 Supplemental Information 4 Comparison of mycobiota between male and female RA patients. Comparison of the Chao1 (A), and Shannon (B) index of two groups; Principal coordinate analysis (PCoA) plots of individual fungal microbiota based on unweighted (C) Unifrac distances in the male and female RA patients; (D) The PLS-DA score plots showing model discrimination between male and female RA patients; (E) The VIP plot indicating the most discriminating fungal taxa in the descending order of importance. The colored boxes on the right indicate the relative amount of the corresponding taxa in each group.

## Additional Information and Declarations

### Data Availability

The following information was supplied regarding data availability: The sequence data are available in the GenBank Sequence Read Archive: PRJNA735021.
