# Supplementary material for: Alterations of gut fungal microbiota in patients with rheumatoid arthritis
Source: PeerJ. 2022 Mar 1;10:e13037. doi: 10.7717/peerj.13037 (PMC8896017; doi:10.7717/peerj.13037)
Supplement: Supplemental Information 2 — Comparison of the Chao1 (A), and Shannon (B) index of two groups; Principal coordinate analysis (PCoA) plots of individual fungal microbiota based on unweighted (C) Unifrac distances in the RA patients and the healthy controls; (D) The PLS-DA score plots showing model discrimination between RA and HC groups; (E) The VIP plot indicating the most discriminating fungal taxa in the descending order of importance. The colored boxes on the right indicate the relative amount of the corresponding taxa in each group. [file peerj-10-13037-s002.docx]

**Figure S1**


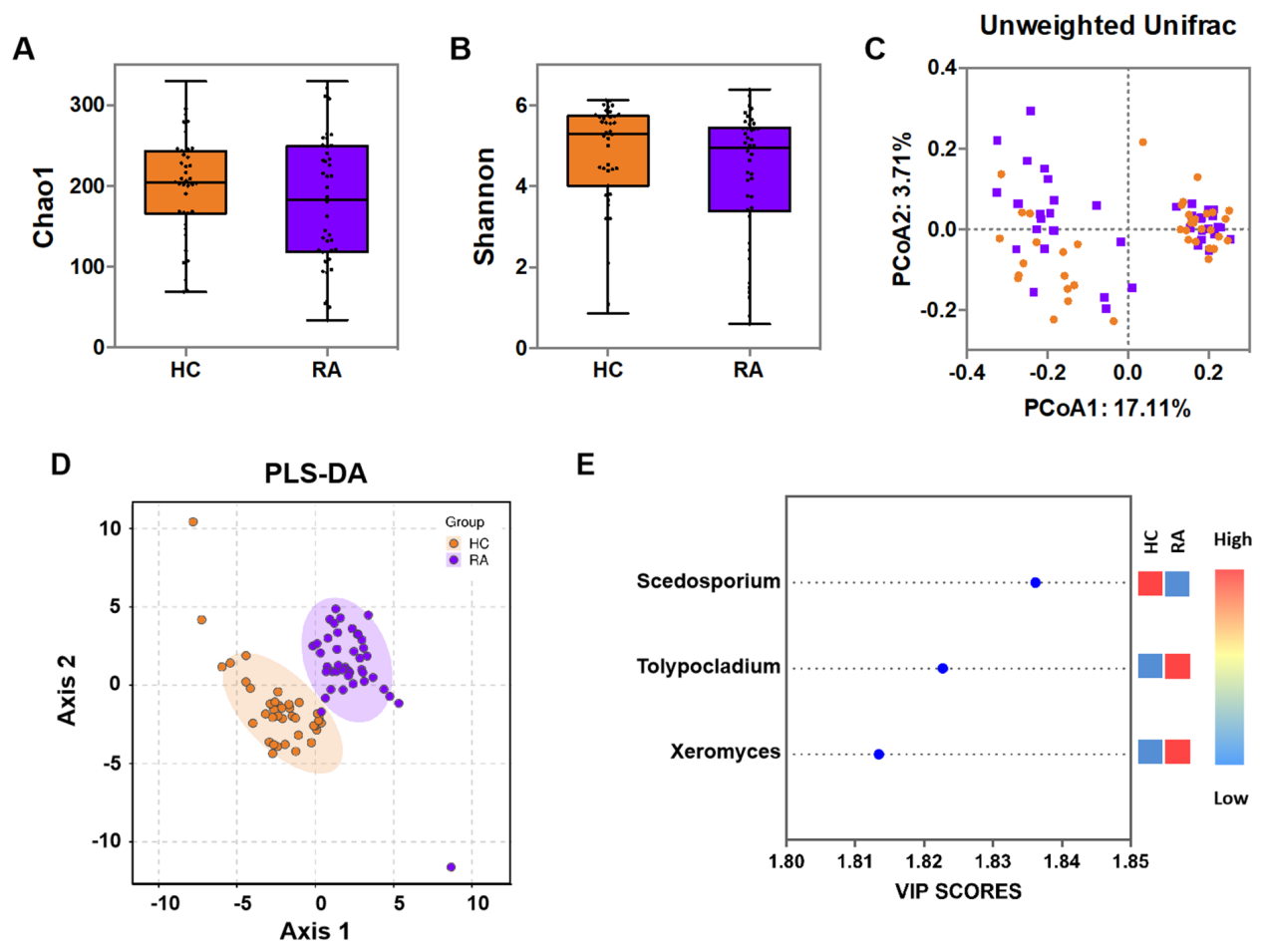


**Figure S1** Comparasion of mycobiota between RA patients and health controls. Comparison of the Chao1 (**A**), and Shannon (**B**) index of two groups; Principal coordinate analysis (PCoA) plots of individual fungal microbiota based on unweighted (**C**) Unifrac distances in the RA patients and the healthy controls; (D) The PLS-DA score plots showing model discrimination between RA and HC groups; (E) The VIP plot indicating the most discriminating fungal taxa in the descending order of importance. The colored boxes on the right indicate the relative amount of the corresponding taxa in each group.
