# Supplementary material for: Alterations of gut fungal microbiota in patients with rheumatoid arthritis
Source: PeerJ. 2022 Mar 1;10:e13037. doi: 10.7717/peerj.13037 (PMC8896017; doi:10.7717/peerj.13037)
Supplement: Supplemental Information 3 — Comparison of the Chao1 (A), and Shannon (B) index of two groups; Principal coordinate analysis (PCoA) plots of individual fungal microbiota based on unweighted (C) Unifrac distances in the RA patients aged less than 60 and over 60 years; (D) The PLS-DA score plots showing model discrimination between RA patients aged less than 60 and over 60 years; (E) The VIP plot indicating the most discriminating fungal taxa in the descending order of importance. The colored boxes on the right indicate the relative amount of the corresponding taxa in each group. [file peerj-10-13037-s003.docx]

**Figure S2**


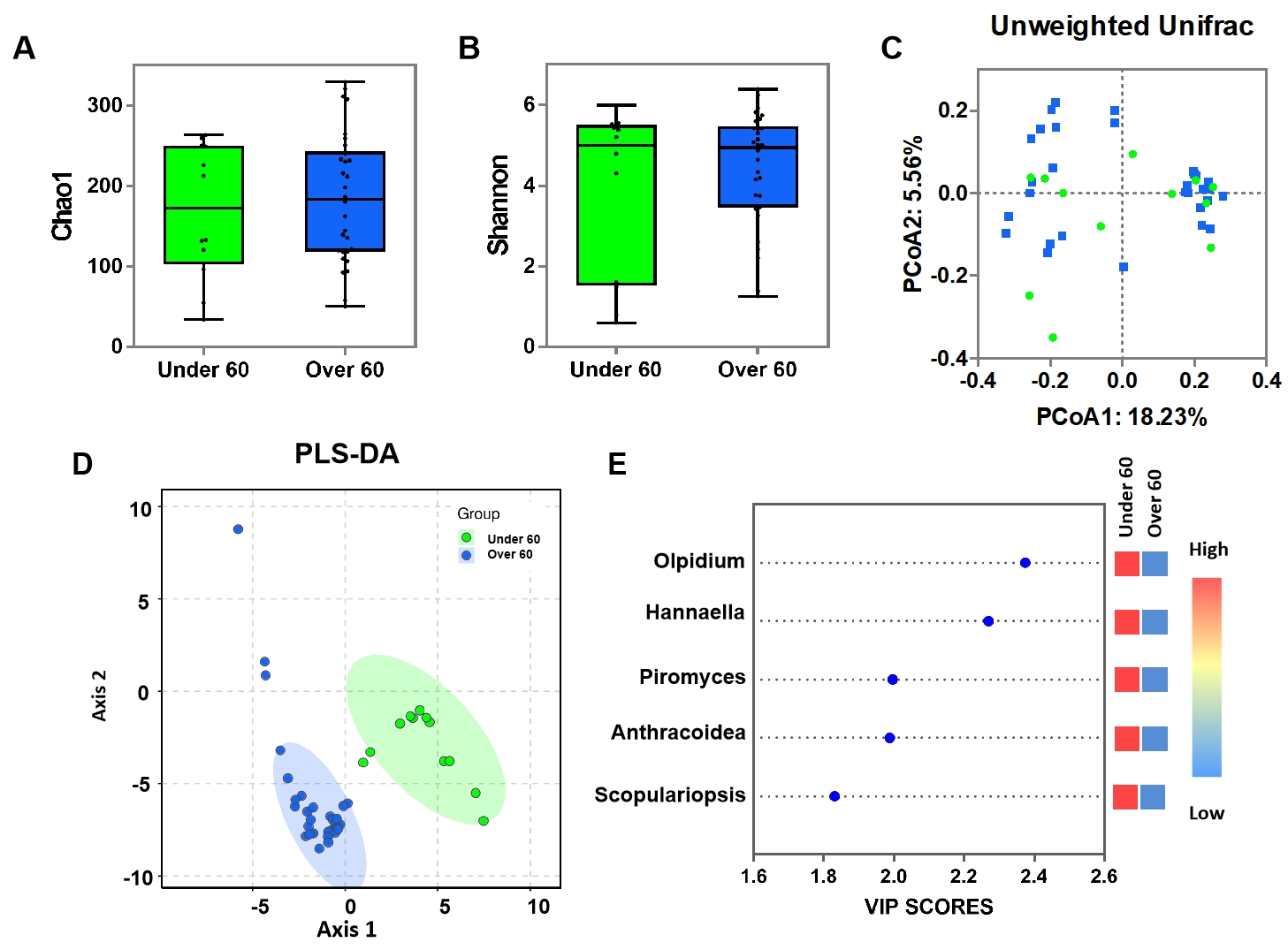


**Figure S2** Comparasion of mycobiota between RA patients aged less than 60 and those over 60 years of age. Comparison of the Chao1 (**A**), and Shannon (**B**) index of two groups; Principal coordinate analysis (PCoA) plots of individual fungal microbiota based on unweighted (**C**) Unifrac distances in the RA patients aged less than 60 and over 60 years; (**D**) The PLS-DA score plots showing model discrimination between RA patients aged less than 60 and over 60 years; (**E**) The VIP plot indicating the most discriminating fungal taxa in the descending order of importance. The colored boxes on the right indicate the relative amount of the corresponding taxa in each group.
