# Supplementary material for: Alterations of gut fungal microbiota in patients with rheumatoid arthritis
Source: PeerJ. 2022 Mar 1;10:e13037. doi: 10.7717/peerj.13037 (PMC8896017; doi:10.7717/peerj.13037)
Supplement: Supplemental Information 4 — Comparison of the Chao1 (A), and Shannon (B) index of two groups; Principal coordinate analysis (PCoA) plots of individual fungal microbiota based on unweighted (C) Unifrac distances in the male and female RA patients; (D) The PLS-DA score plots showing model discrimination between male and female RA patients; (E) The VIP plot indicating the most discriminating fungal taxa in the descending order of importance. The colored boxes on the right indicate the relative amount of the corresponding taxa in each group. [file peerj-10-13037-s004.docx]

**Figure S3**

**
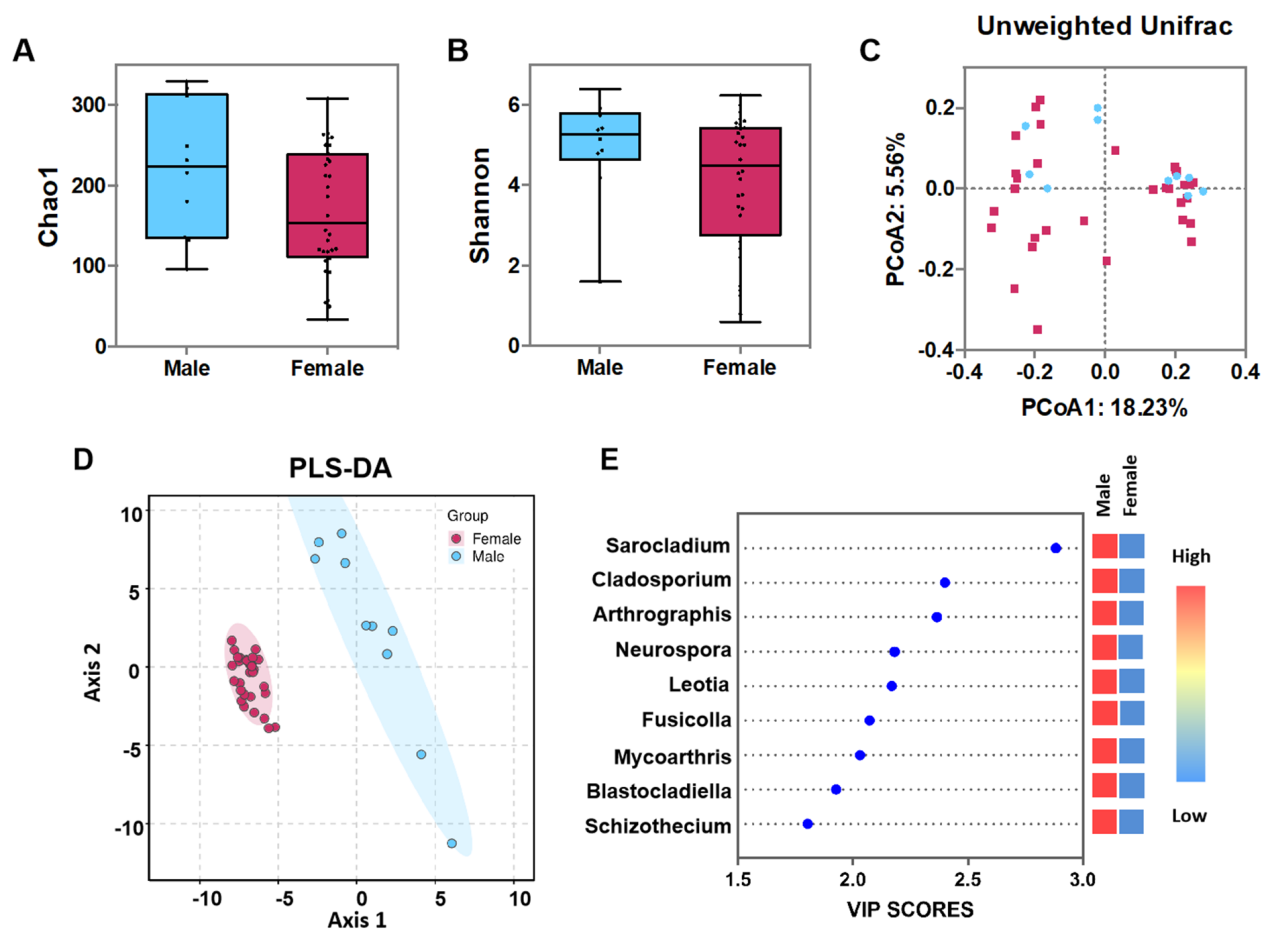
**

**Figure S3** Comparasion of mycobiota between male and female RA patients. Comparison of the Chao1 (**A**), and Shannon (**B**) index of two groups; Principal coordinate analysis (PCoA) plots of individual fungal microbiota based on unweighted (**C**) Unifrac distances in the male and female RA patients; (**D**) The PLS-DA score plots showing model discrimination between male and female RA patients; (**E**) The VIP plot indicating the most discriminating fungal taxa in the descending order of importance. The colored boxes on the right indicate the relative amount of the corresponding taxa in each group.
